# Dedicated versus non-dedicated transcatheter valves for pure native aortic regurgitation: a single-centre experience

**DOI:** 10.1007/s12471-026-02048-4

**Published:** 2026-05-12

**Authors:** Lucas Uchoa de Assis, Antigone Kostea, Andrea Mariani, Giulio M. Mondellini, Mark M. P. van den Dorpel, Isabella Kardys, Joost Daemen, Rutger-Jan Nuis, Nicolas M. Van Mieghem

**Affiliations:** 1 Department of Cardiology, Thoraxcenter, Cardiovascular Institute, Erasmus University Medical Center, Rotterdam, The Netherlands; 2 Department of Advanced Biomedical Sciences, University of Naples Federico II, Naples, Italy

**Keywords:** Transcatheter Aortic Valve Replacement, Aortic Valve Implantation, Aortic Valve Insufficiency, Heart Valve Prosthesis, Treatment Outcome, Pacemaker

## Abstract

**Background:**

Aortic regurgitation (AR) is the third most common valvular heart disease in the Western world. Management of high-surgical-risk patients is challenging. The absence of calcifications in the aortic valve presents anchoring challenges for transcatheter aortic valve implantation (TAVI) in patients with severe native AR.

The Trilogy device is a dedicated transcatheter heart valve for the treatment of AR and anchors by clipping onto the native aortic valve leaflets. Real-world data comparing dedicated vs. non-dedicated THVs remains scarce.

**Methods:**

We evaluated 42 consecutive high- or prohibitive-surgical-risk patients with pure native AR who underwent transfemoral TAVI. Twenty-one patients received the Trilogy device, and 21 received non-dedicated transcatheter valves (THV). Co-primary endpoints were device success and early safety at 30 days. Secondary endpoints included valve embolisation, residual moderate-to-severe AR, new pacemaker implantation, mortality, and length of hospital stay.

**Results:**

Device success was significantly higher with dedicated THVs (100% vs. 61.9%; *p* = 0.002). Early safety was comparable (71.4% vs. 61.9%; *p* = 0.513). There were 4 valve embolisations (9.5%) and two cardiovascular deaths (4.8%) in the non-dedicated group. Residual moderate-to-severe AR was absent in the dedicated cohort and occurred in 2 of 21 non-dedicated cases. Overall, new permanent pacemaker implantation occurred in 19%, with no significant difference between the 2 cohorts (*p* = 1.00). Median hospital stay was significantly shorter in the dedicated cohort (2 vs. 5 days; *p* = 0.015).

**Conclusion:**

TAVI with the Trilogy valve system had higher device success as compared to non-dedicated valves and should become the preferred transcatheter strategy for patients with severe native AR at high-operative risk.

**Supplementary Information:**

The online version of this article (10.1007/s12471-026-02048-4) contains supplementary material, which is available to authorized users.

## What’s new?

- In high-risk patients with native pure aortic regurgitation, the dedicated Trilogy valve, with its unique leaflet anchoring properties, achieved 100% device success in non-calcified anatomy, compared to 62% device success with non-dedicated, off-label transcatheter heart valves.
- The dedicated Trilogy valve was associated with favorable 30-day clinical outcomes and shorter hospital stay.
- The incidence of new permanent pacemaker implantation after TAVI for pure aortic regurgitation remains notably high (19%) for both dedicated and non-dedicated devices, highlighting the need for further investigation.

## Introduction

Aortic regurgitation (AR) is the third-most common valvular heart disease in the Western world, with its prevalence and burden rising with an ageing population [1–4]. Surgical aortic valve replacement (SAVR) is the recommended treatment for severe AR [5]. However, less-invasive transcatheter strategies are needed to treat patients with native AR at high-to-prohibitive operative risk [6, 7]. While transcatheter aortic valve implantation (TAVI) emerged as a transformative, minimally invasive therapy for calcific severe aortic stenosis (AS), its “off-label” use in patients with pure AR proved to be more challenging. The lack of annular calcification, high stroke volumes, and large annuli may cause anchoring issues, with a 12% risk of device embolisation and a 10–20% risk of moderate-to-severe residual paravalvular leakage (PVL) [6, 8]. The Trilogy valve system (JenaValve Technology, Irvine, CA, USA) is a supra-annular self-expanding transcatheter heart valve (THV) system that received CE-Mark for AR treatment in 2021. A tri-leaflet clipping mechanism anchors the frame to the native cusps, independent of calcium. Early reports on TAVI with the Trilogy device for pure AR are encouraging [9]. There is still limited real-world outcome data on the use of dedicated and non-dedicated THVs for pure AR. We aim to describe device success and early safety of TAVI for pure AR with dedicated and non-dedicated THVs.

## Methods

### Study population and design

Consecutive patients undergoing TAVI for pure native AR at the Erasmus Medical Center between 2012 and 2025 were included. A multidisciplinary heart valve team evaluated all patients. Selection for TAVI was based on high/prohibitive surgical risk and anatomical suitability. Patients with mixed aortic disease and moderate to severely calcified aortic leaflets were excluded. The need for individual patient consent was waived by the ethics committee, and the study was conducted in accordance with the Declaration of Helsinki.

### Procedural planning and strategy

Transthoracic echocardiography and computed tomography (CT) were performed to evaluate anatomy, calcification, annular dimension, mechanism of AR, and transfemoral access suitability. AR severity was assessed according to the American Society of Echocardiography guidelines for native AR [10].

Patients were categorized based on the type of THV implanted. The Trilogy valve system was the only dedicated THV for AR treatment in our centre and was introduced in 2019. Non-dedicated THVs were designed for treating calcific AS and were used off-label for AR treatment. Device sizing was per the manufacturer’s instruction for use (IFU) for the Trilogy device. Valve oversizing was calculated relative to the annulus, using the perimeter-derived diameter for self-expanding THV and the area-derived diameter for balloon-expandable valves. Conversely, for non-dedicated THVs, more oversizing than recommended per the manufacturer’s IFU was common to aim for better anchoring in the absence of aortic valve calcifications.

### Endpoint definitions

Device success and early safety at 30 days were defined according to Valve Academic Research Consortium‑3 (VARC-3) criteria [11]. Device success was defined as the composite of technical success, absence of device-related mortality or reintervention, and intended valve performance (mean gradient < 20 mm Hg, peak velocity < 3 m/s, Doppler velocity index ≥ 0.25, and less than moderate residual AR). Early safety was a composite endpoint including all-cause mortality, stroke, VARC type III-IV bleeding, major vascular complications, stage 3 acute kidney injury, permanent pacemaker (PPM) implantation, and valve-related surgical or interventional procedures.

Secondary endpoints included the individual components of the co-primary endpoints, cardiovascular mortality, transient ischemic attack, hospital readmission for heart failure, and length of hospital stay (LOS).

### Statistical analysis

Continuous variables were reported as mean ± standard deviation or median with 25th and 75th percentiles, depending on distribution. Categorical variables were expressed as frequencies and percentages. Comparisons between dedicated and non-dedicated THV groups were performed using the Student’s *t*-test or Mann-Whitney U‑test, and the chi-square test or Fisher’s exact test, as appropriate. Univariable logistic regression was used to assess predictors of PPM, with results reported as odds ratios and 95% confidence intervals. A two-tailed *p*-value < 0.05 was considered statistically significant. Statistical analyses were performed using IBM SPSS version 28.0.1.0 (IBM Corp., Armonk, NY, USA).

## Results

### Baseline characteristics

This study included 42 patients undergoing TAVI for pure AR. An equal number of patients received a dedicated THV (*n* = 21) and a non-dedicated THV (*n* = 21) (Tab. 1**)**. Most patients were women (69%). Mean age was 72.4 ± 10.1 years. Median Society of Thoracic Surgeons Predicted Risk of Mortality (STS-PROM) score was 2.9 [1.9–4.8] %. Patients in the dedicated THV cohort were older (75.5 ± 7.6 vs. 69.4 ± 11.6 years, *p* = 0.05) and had a significantly higher baseline left ventricular (LV) ejection fraction (46.0 ± 11.7%) compared to the non-dedicated cohort (37.7 ± 12.8%, *p* = 0.02).

**Table 1:** Baseline characteristics of patients undergoing TAVI for pure aortic regurgitation.

	All patients (*n* = 42)	Dedicated THV (*n* = 21)	Non-dedicated THV (*n* = 21)	*p*-value
*Demographics*				
Age, years	72.4 ± 10.1	75.5 ± 7.6	69.4 ± 11.6	0.05
Male, *n* (%)	13 (31)	5 (24)	8 (38)	0.33
Body mass index, kg/m^2^	23.5 ± 4.3	23.0 ± 3.1	24.0 ± 5.3	0.47
STS score, %	2.9 [1.9–4.8]	3.4 [2.1–4.2]	2.3 [1.9–6.2]	0.79
*NYHA class*				0.80
NYHA I, *n* (%)	2 (5)	1 (5)	1 (5)	
NYHA II, *n* (%)	9 (21)	5 (24)	4 (19)	
NYHA III, *n* (%)	28 (67)	14 (67)	14 (67)	
NYHA IV, *n* (%)	3 (7)	1 (5)	2 (10)	
*Echocardiography*				
LVEF, %	41.9 ± 12.8	46.0 ± 11.7	37.7 ± 12.8	0.02
LVEDD, mm	56.6 ± 11.5	54.9 ± 9.4	58.2 ± 13.6	0.34
Peak aortic velocity, m/s	2.04 ± 0.78	2.21 ± 0.78	1.86 ± 0.76	0.16
RV systolic pressure, mm Hg †	38 [27–48]	40 [36–48]	36 [23–45]	0.43
*Comorbidities, n (%)*				
Hypertension, *n* (%)	27 (64%)	13 (62%)	14 (67%)	0.75
Diabetes, *n* (%)	2 (5)	2 (10)	0	0.15
Peripheral artery disease, *n* (%)	8 (19)	5 (24)	3 (14)	0.42
Prior myocardial infarction, *n* (%)	8 (19)	2 (10)	6 (29)	0.24
Prior PCI, *n* (%)	9 (21)	3 (14)	6 (29)	0.26
Prior CABG, *n* (%)	3 (7)	1 (5)	2 (10)	0.55
Prior pacemaker/AICD, *n* (%)	9 (21)	4 (19)	5 (24)	0.71
Atrial fibrillation, *n* (%)	10 (24)	6 (29)	4 (19)	0.46
Prior stroke, *n* (%)	3 (7)	2 (10)	1 (5)	0.55
Prior TIA, *n* (%)	5 (12)	5 (24)	0	**0.02**
Pulmonary hypertension, *n* (%)	4 (10)	4 (19)	0	**0.04**
Chronic kidney disease, *n* (%)	20 (48)	8 (38)	12 (57)	0.26
Prior kidney transplant, *n* (%)	3 (7)	2 (10)	1 (5)	0.55
Prior LVAD, *n* (%)	3 (7)	1 (5)	2 (10)	0.55
Urgent procedure, *n* (%)	3 (7)	1 (5)	2 (10)	0.55

†RVSP values were available in 29/42 patients

Abbreviations: *AICD* automatic implantable cardioverter defibrillator, *BMI* body mass index, *CABG* coronary artery bypass grafting, *IQR* interquartile range, *LVEF* left ventricular ejection fraction, *LVEDD* left ventricular end-diastolic diameter, *PCI* percutaneous coronary intervention, *STS* Society of Thoracic Surgeons risk score, *NYHA* New York Heart Association, *TIA* transient ischemic attack, *LVAD* left ventricular assist device

### CT characteristics

Aortic annular dimensions were similar, with mean annular areas of 472.9 ± 84.1 mm^2^ in the dedicated THV group and 488.7 ± 108.3 mm^2^ in the non-dedicated group (*p* = 0.60) **(**Tab. 2**)**. Ascending aortic diameter was significantly larger in the dedicated THV group compared to the non-dedicated group (38.6 ± 5.7 mm vs.34.0 ± 6.3 mm, *p* = 0.02). The median Agatston score was low and similar for the dedicated and non-dedicated THV cohorts (dedicated: 148 [78–804] AU vs. non-dedicated: 80 [0–970] AU; *p* = 0.57).

**Table 2:** CT and procedural characteristics

	All patients (*n* = 42)	Dedicated (*n* = 21)	Non-dedicated (*n* = 21)	*p*-value
*Procedural planning*				
Annular area, mm	480.3 ± 96.1	472.9 ± 84.1	488.7 ± 108.3	0.62
Annular perimeter, mm	78.2 ± 7.8	77.7 ± 7.1	78.6 ± 8.6	0.72
Area-derived diameter, mm	24.6 ± 2.5	24.4 ± 2.2	24.8 ± 2.7	0.64
LVOT diameter, mm	24.6 ± 2.6	24.6 ± 2.4	24.6 ± 2.8	0.94
Sinus of Valsalva diameter, mm	35.7 ± 4.6^1^	35.4 ± 4.7	36.0 ± 4.7	0.69
Ascending aortic diameter, mm	36.2 ± 6.4^2^	38.6 ± 5.7	34.0 ± 6.3	**0.02**
Agatston calcium score, AU ‡	147.5 [0–840]	148 [78–804]	80 [0–970]	0.57
*Valve type*				
JenaValve, *n* (%)	21 (50.0)	21 (100)	0 (0.0)	
CoreValve/Evolut, *n* (%)	5 (11.9)		5 (23.8)	
Edwards Sapien 3, *n* (%)	4 (9.5)		4 (19.0)	
Lotus/Lotus Edge, *n* (%)	3 (7.1)		3 (14.3)	
Portico, *n* (%)	1 (2.4)		1 (4.8)	
Acurate Neo/Prime, *n* (%)	6 (14.3)		6 (28.6)	
Myval Octacor, *n* (%)	2 (4.8)		2 (9.5)	
*Oversizing*				
Self-expanding valves —Perimeter oversizing (%)†	13.2 ± 6.1	15.5 ± 4.7	10.1 ± 6.5	**<** **0.01**
Balloon-expandable—Area oversizing (%)*	9.9 ± 3.8	–	9.9 ± 3.8	**–**
*Procedural characteristics*				
General anaesthesia	6 (14.3)	0	6 (28.6)	<0.01
Cerebral protection device use	1 (2.4)	0	1 (4.8)	0.31
Rapid ventricular pacing	34 (81.0)	15 (71.4)	19 (90)	0.12
Contrast volume (mL)	108.57 ± 44.51	112.0 ± 36.07	105.24 ± 50.88	0.63
Fluoroscopy time (min) *	14.5 [11.2–20.1]	15.4 [11.6–21.1]	12.5 [6.8–22.6]	0.18
Post-dilatation performed	5 (11.9)	2 (9.5)	3 (14.3)	0.62

^‡^Based on *n* = 30

†Perimeter oversizing was calculated as the ratio of annulus perimeter to the valve waist perimeter for all self-expanding valves

^*^Area oversizing was calculated as the ratio of annulus area to the valve area for balloon-expandable valves

*LVOT* left ventricular outflow

### Procedural characteristics

Six off-label platforms were used in the non-dedicated group: Acurate Neo/Prime (*n* = 6), CoreValve/Evolut (*n* = 5), Sapien3 (*n* = 4), Lotus (*n* = 3), Myval (*n* = 2), and Portico (*n* = 1). Among self-expanding valves, perimeter-derived oversizing was significantly greater in the dedicated group compared with non-dedicated self-expanding valves (15.5 ± 4.7% vs. 10.1 ± 6.5%, *p* = < 0.01). Area-derived oversizing was 9.9. ± 3.8% with balloon-expandable valves. Post-dilatation was performed in 11.9% of patients, with no significant difference between the groups (*p* = 0.62). Rapid pacing was performed in all patients in the non-dedicated TAVI group, except for the two patients who received a Lotus valve, and 71% (*n* = 15) of the dedicated group.

### Clinical outcomes

**Fig. 1:**
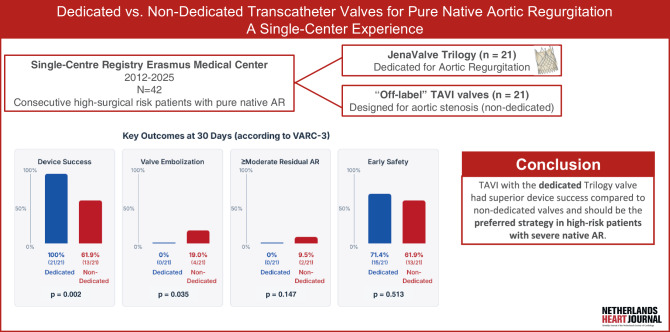
Infographic

At 30 days, two patients (4.8%) died in the non-dedicated THV group, both classified as cardiovascular deaths (Tab. 3). Device success was achieved in all patients of the dedicated THV group vs. 62% of patients in the non-dedicated cohort (*p* = 0.002) (Fig. 1). Early safety success was observed in 28 patients (66.7%), with no significant difference between groups (71.4%vs.61.9%, *p* = 0.513).

**Table 3:** Clinical outcomes at 30 days following TAVI for pure native aortic regurgitation

Outcome	Total (*n* = 42)	Dedicated (*n* = 21)	Non-dedicated (*n* = 21)	*p*-value
All-cause mortality, *n* (%)	2 (4.8)	0 (0.0)	2 (9.5)	0.147
Cardiovascular mortality, *n* (%)	2 (4.8)	0 (0.0)	2 (9.5)	0.147
Device success, *n* (%)	34 (81.0)	21 (100.0)	13 (61.9)	0.002
Positioning success, *n* (%)	38 (90.5)	21 (100.0)	17 (81.0)	0.035
Post-procedural AR ≥ moderate, *n* (%)	2 (4.8)	0 (0.0)	2 (9.5)	0.147
Valve embolization, *n* (%)	4 (9.5)	0 (0.0)	4 (19.0)	0.035
Conversion to open surgery, *n* (%)	2 (4.8)	0 (0.0)	2 (9.5)	0.147
Early safety, *n* (%)	28 (66.7)	15 (71.4)	13 (61.9)	0.513
Stroke, *n*	0	0	0	–
TIA, *n* (%)	1 (2.4)	1 (4.8)	0 (0.0)	0.311
Major vascular complications, *n* (%)	2 (4.8)	0 (0.0)	2 (9.5)	0.147
Minor vascular complications, *n* (%)	3 (7.1)	2 (9.5)	1 (4.8)	0.549
Major bleeding (VARC-3 ≥ Type 2), *n* (%)	6 (14.3)	3 (14.3)	3 (14.3)	1.000
Acute Kidney Injury stage 3 or 4, *n* (%)	4 (10)	2 (10)	2 (10)	1.000
Post-procedural dialysis, *n* (%)	1 (2.4)	0 (0.0)	1 (4.8)	0.311
New permanent pacemaker, *n* (%)*	6 (19.4)	3 (17.6)	3 (21.4)	1.000
Cardiac reintervention, *n* (%)	0 (0.0)	0 (0.0)	0 (0.0)	–
Length of hospital stay, median [IQR], days †	3 [2–8]	2 [1–3]	5 [3–10]	0.015
NYHA class at 6 weeks, *n* (%)				0.630
NYHA I	15 (47)	7 (47)	8 (47)	
NYHA II	14 (44)	8 (53)	6 (35)	
NYHA III	3 (9)	0	3 (18)	
NYHA IV	0	0	0	

Abbreviations: *AR* aortic regurgitation, *IQR* interquartile range, *NYHA* New York Heart Association, *TAVI* transcatheter aortic valve implantation, *THV* transcatheter heart valve, *VARC‑3* Valve Academic Research Consortium‑3

†Mann-Whitney U test due to non-normal distribution

*Percentage of pacemaker naïve patients

Valve embolisation did not happen in the dedicated THV cohort and occurred in 4 patients (9.5%) in the non-dedicated cohort (*p* = 0.035). Two of these events required emergency surgical conversion.

In one patient, a 29 mm SAPIEN3 (3% oversizing) embolised into the LV, necessitating surgical retrieval and valve replacement. In another patient, suboptimal positioning of a 29 mm SAPIEN3 (13% oversizing) led to severe PVL, and a second 29 mm SAPIEN3 was implanted. However, both valves embolised into the LV, and the patient did not survive. One patient experienced gradual embolisation of a 29 mm CoreValve (13% oversizing) into the ascending aorta; this was managed by implanting a second 31 mm CoreValve (TAV-in-TAV). In one patient, the 32 mm Myval (13% oversizing) embolised towards the ascending aorta during deployment and was stabilized by implanting a 34 mm Evolut (TAV-in-TAV). Annular rupture never occurred. Moderate-to-severe AR post-procedure was observed in 2 patients in the non-dedicated group.

PPM implantation was required in 6 patients (19% of pacemaker naïve patients), with three in both groups (*p* = 1.00). No strokes were reported. Major vascular complications occurred in 2 patients (4.8%), both in the non-dedicated group (*p* = 0.147). VARC‑3 type ≥ 2 bleeding occurred in 6 patients (14.3%), equally distributed between both groups. Median LOS was 3 days [2–8], significantly shorter in the dedicated THV group (2[1–3] vs. 5[3–10] days; *p* = 0.015). Device and technical success were achieved in all 3 LVAD patients who received a 26 mm Sapien3 valve (LVAD for destination therapy), 23 mm ACURATE neo2 valve (bridge-to-transplant; transplantation 2 months later), and a Trilogy M‑size valve (destination therapy).

Predictors for PPM implantation included annular perimeter size (OR:2.41, 95% CI:1.12–5.21, *p* < 0.01) and valve size (OR:1.55, 95% CI:1.02–2.34, *p* = 0.02) (See Electronic Supplementary Material [ESM] Tab.S1).

## Discussion

In this 13-year, single-centre experience, we report the largest Dutch series of TAVI for pure native AR to date. The dedicated Trilogy device yielded 100% device success with no deaths, embolisations or ≥ moderate residual AR at 30 days, whereas off-label THVs achieved 62% device success, accounting for both procedure-related deaths (10% of non-dedicated THV cases), four valvular embolisations (19%), and two cases (10%) of ≥ moderate residual AR.

Our results with the Trilogy device are consistent with the current data on dedicated systems. The prospective, multicentre ALIGN-AR trial (Transcatheter aortic valve implantation in patients with high-risk symptomatic native aortic regurgitation; NCT04415047) examined clinical outcomes of the Trilogy device in patients with symptomatic moderate-to-severe AR at high surgical risk. The trial enrolled 700 patients (180 in a pivotal cohort and 520 in continued access) in the US. Technical success was 95%, with an embolisation rate of 1.3% and a 30-day mortality rate of 1.6% [9, 12]. These results were further supported by the European multicentre PURPOSE (Performance of Purpose-Built vs Off-Label Transcatheter Devices for Aortic Regurgitation) registry, which confirmed high technical success (98%) and a low embolisation rate (1%) in 88 pure AR patients treated with the Trilogy device.

The relatively low device success rate after TAVI with non-dedicated devices in our series (62%), with an embolisation rate of 19% and moderate-to-severe PVL in 10%, attests to the unpredictability of non-dedicated THVs in the context of pure AR, with no aortic root calcifications for proper anchoring. Previously, the PANTHEON registry (Performance of Currently Available Transcatheter Aortic Valve Platforms in Inoperable Patients With Pure Aortic Regurgitation of a Native Valve) (*n* = 201) also reported modest outcomes, with a device success rate of 76%, an embolisation rate of 12%, and moderate-to-severe PVL in 10% of cases, corroborating the unmet clinical need for dedicated transcatheter technologies to treat pure AR [8].

A recent meta-analysis including 2162 AR patients confirmed these challenges. Non-dedicated device implantations made up 45% (*n* = 969) of the analysis and showed substantially lower device success compared with Trilogy/J-valve cases (82%vs. 93%), higher rates of ≥ moderate residual AR (5%vs. 3%), increased valve embolisation (8%vs. 2%), and a higher 30-day mortality rate (9%vs. 3%) [13].

In our study, oversizing was higher with the Trilogy device compared to other self-expanding devices. The oversizing with the Trilogy device was in accordance with the IFU, which recommends 10–20% oversizing, whereas for non-dedicated self-expanding valves, often 10–15% is advised [14]. Excessive oversizing (> 20%) should be avoided, as it may increase the risk of annular rupture [14, 15]. Incremental oversizing is also associated with a higher incidence of conduction disorders and need for PPM after TAVI for severe AS.

PPM implantation (*n* = 6) was the main driver for early safety endpoint failure. The pacemaker rate in our study is similar to rates reported in ALIGN-AR and PURPOSE (24%), and remains clinically relevant, as it exceeds the pacemaker rates typically seen in contemporary TAVI for AS and SAVR [9, 16]. In our cohort, a larger perimeter was associated with a higher rate of PPM, whereas oversizing was not. Similarly, in ALIGN-AR, larger annular dimensions but not valve oversizing predicted the need for PPM implantation [17]. Conversely, in another retrospective registry of 141 patients who underwent TAVI for pure AR, oversizing and annular dimensions were not associated with the need for PPM [18]. The mechanism of conduction injury after TAVI for pure AR remains elusive. The effects of transcatheter valve properties and implantation depths, LV volume overload that may render the conduction system more vulnerable, and the absence of calcifications that may otherwise protect against pressure trauma need further study [13].

Three LVAD patients with AR underwent successful TAVI. AR in LVAD patients may arise from mechanical stress, leading to valvular incompetence and aortic dilatation [19]. Moderate-to-severe AR occurred in up to 30% of early-generation LVAD patients within 1 year, but HeartMate3 support has reduced this incidence to 3–4% [20]. Still, progressive AR remains a vexing issue with contemporary LVADs. LVAD Flow should be reduced during the implant procedure to mitigate suction effects and risk of THV migration. Additionally, after TAVI, the lower flow and valve opening raise the risk of leaflet thrombosis.

### Clinical implications

SAVR remains the standard of care for treatment of severe, pure AR. However, undertreatment is prevalent. One-third of patients with an indication for SAVR are not referred due to perceived high surgical risk, and these patients experience a 2-year mortality rate exceeding 20% [21–24]. Contemporary data support earlier referral to a Heart Valve Team and reassessment of perceived surgical futility [22, 24]. Our data further reinforce the clinical merits of TAVI with a dedicated platform in AR patients deemed at high surgical risk by the Heart Valve Team. The 2025 ESC/EACTS guidelines for the management of valvular heart disease granted a class IIb recommendation for TAVI with dedicated devices for the treatment of severe AR [5].

While availability is currently restricted, referral to centres with access to dedicated devices seems reasonable and preferable over the use of non-dedicated systems. A further important limitation remains the sizing matrix, which is restricted to annular diameters between 21–27 mm.

### Future developments

The ARTIST (Aortic Regurgitation Trial Investigating Surgery Versus Trilogy; NCT06608823) trial is an ongoing randomised controlled trial comparing the Trilogy device with SAVR and aims to include 1016 low-to-intermediate risk AR patients. In addition, the JENA-VAD (JenaValve ALIGN-AR LVAD Registry; NCT06594705) is an ongoing registry that aims to assess clinical outcomes in 50 LVAD patients undergoing TAVI with the Trilogy device.

Other devices are under development. The J‑Valve (JC Medical Inc., Burlingame, CA, USA) consists of a self-expanding nitinol frame and three U‑shaped anchor rings that grasp the native leaflets. An early feasibility study in 25 patients demonstrated 92% procedural success without device embolisation and 1 (non-cardiovascular) death at 30 days [25, 26]. The JOURNEY trial (J-Valve to Treat Aortic Regurgitation via Transcatheter Therapy; NCT06455787) aims to enroll 194 patients to further assess the efficacy and safety of the J‑valve.

The Hanchor valve (Healing Medical Technology, Shanghai, China) is a balloon-expandable, dedicated THV featuring a nitinol anchor element [27]. In early results from the multicentre HAVE-AR trial (*n* = 128) in China, procedural success was 96%, with 2% embolisation and no moderate-to-severe AR at 30-days [27]. The Cusper (Cuspa LTD, Nazareth, Israel) is a transcatheter aortic repair system that consists of a pericardial sac attached to a nitinol clip, which is delivered transfemorally and clipped onto the aortic leaflet. The sac fills the central coaptation gap, directly addressing central AR [28].

### Limitations

Several limitations must be acknowledged. The cohort is single-centre and modest, which may limit statistical power and external validity. However, our results mirror those of other registries and meta-analyses. The retrospective design holds timing and selection bias. The non-dedicated cohort, spanning 13 years, is a historical comparator. It comprises a heterogeneous mix of devices, including balloon-expandable and early-generation valves. In our early experience, only non-dedicated valves were used. Consequently, the observed differences in outcomes also reflect procedural learning and the evolution of device technology. Additionally, the restricted sizing matrix of the Trilogy device precluded the treatment of very large annular anatomies in the dedicated group.

In-house expertise was used for the assessment of echocardiographic and CT images and for adjudication of clinical events.

## Conclusions

TAVI with the dedicated Trilogy device was associated with higher device success compared to non-dedicated valves and should become the preferred transcatheter strategy for patients with severe native AR at high-operative risk.

## Supplementary Information

ESM1: Supplementary material 1

## Data Availability

The datasets generated during and/or analyzed during the current study are available from the corresponding author on reasonable request.
