# Supplementary material for: Dedicated versus non-dedicated transcatheter valves for pure native aortic regurgitation: a single-centre experience
Source: Neth Heart J. 2026 May 12;34(6):206–15. doi: 10.1007/s12471-026-02048-4 (PMC13216396; doi:10.1007/s12471-026-02048-4)
Supplement: Supplementary file 1 — ESM1: Supplementary material 1 [file 12471_2026_2048_MOESM1_ESM.docx]

| **Variable** | **Odds Ratio (OR)** | **95 CI** | **p-value** |
| --- | --- | --- | --- |
| LVEDD (mm) | 1.02 | 0.93 – 1.12 | 0.70 |
| LVOT diameter (mm) | 1.47 | 0.97 – 2.23 | 0.05 |
| Perimeter (mm) | 2.41 | 1.12 – 5.21 | **< 0.001** |
| Valve size implanted (mm) | 1.55 | 1.02 – 2.34 | **0.02** |
| Perimeter oversizing (%) | 0.91 | 0.76 – 1.10 | 0.29 |

**Supplemental Table 1. Univariate Logistic regression analysis for predictors of new pacemaker implantation.**

Abbreviations: CI confidence interval, LVEDD left ventricular end-diastolic diameter, LVOT left ventricular outflow tract, OR odds ratio, PPM permanent pacemaker.
